# MicroRNA-145 is involved in endothelial cell dysfunction and acts as a promising biomarker of acute coronary syndrome

**DOI:** 10.1186/s40001-020-00403-8

**Published:** 2020-03-17

**Authors:** Shanshan Wu, Huijuan Sun, Bin Sun

**Affiliations:** Department of Emergency, Yidu Central Hospital of Weifang, No. 4138, South Linglongshan Road, Weifang, 262500 Shandong China

**Keywords:** Acute coronary syndrome, MicroRNA-145, Diagnosis, Vascular endothelial cell, Inflammation

## Abstract

**Background:**

Acute coronary syndrome (ACS) is a serious type of cardiovascular diseases. This study aimed to investigate the expression patterns and clinical value of microRNA-145 (miR-145) in ACS patients, and further uncover the function of miR-145 in ACS rats.

**Methods:**

Quantitative real-time PCR was used to estimate the expression of miR-145. Diagnostic value of miR-145 was evaluated, and its correlation with endothelial injury marker (vWF and H-FABP) and pro-inflammatory cytokines (IL-6 and TNF-α) was analyzed. Coronary artery ligation was adopted to construct the ACS rat model, and the effects of miR-145 on endothelial injury, inflammation and vascular endothelial cells (VECs) biological function were examined.

**Results:**

Downregulated expression of miR-145 was found in the ACS serum samples compared with the healthy controls. The expression of miR-145 was proved to be a diagnostic biomarker and negatively correlated with vWF, H-FABP, IL-6 and TNF-α. The similar serum expression trends of miR-145 in ACS patients were also observed in the ACS rats, and the overexpression of miR-145 could decrease the elevated vWF, H-FABP, IL-6 and TNF-α in the animal model. Moreover, the upregulation of miR-145 in VECs led to promoted proliferation and migration. The bioinformatics prediction data and luciferase report results indicated that FOXO1 was a direct target of miR-145.

**Conclusions:**

In conclusion, it was hypothesized that serum decreased expression of miR-145 may serve as a potential diagnostic biomarker in ACS patients. Overexpression of miR-145 may improve the endothelial injury and abnormal inflammation through targeting FOXO1, indicating that miR-145 serves as a candidate therapeutic target of ACS.

## Background

Acute coronary syndrome (ACS) is a syndrome in coronary artery disease (CAD) and characterized by a significant decrease in the blood flow in coronary arteries [1]. According to the statistics, ACS is one of the leading causes of global cardiovascular disease-related mortality and disability [2]. ACS consists of two major types based on the presence or absence of myocardial cell necrosis: acute myocardial infarction (AMI) and unstable angina (UA) [3]. The most obvious clinical manifestation of ACS is chest pain, which usually radiates to the angle of the jaw or left shoulder and is accompanied by nausea and sweating [4]. It is reported that ACS occurs mainly due to the thrombosis caused by the rupture of atherosclerotic plaques [5]. Although some processes in the clinical approach have been achieved, the outcomes of patients suffering ACS remain dismal [6]. During the development of ACS, the dysfunction of vascular endothelial cells (VECs) and the impairment in inflammation are considered as two importance events that involved in disease pathogenesis [7]. Thus, some therapeutic strategies against these two pathological events have been highlighted in ACS treatment.

To improve the endothelial injury and balance the inflammatory response in ACS, this study presented an investigation about the protective role of microRNA-145 (miR-145) in this disease. MicroRNAs (miRNAs) are a class of small noncoding RNAs with important regulatory roles in various cellular processes, such as cell proliferation, migration, invasion, differentiation, and cell apoptosis [1, 8]. In addition, the clinical significance of miRNAs also attracts increasing attention for the diagnosis and prognosis of various human diseases [3]. In ACS, some miRNAs with aberrant expression have also been identified as biomarkers and associated with the disease progression [4]. For example, miR-941 has been determined as a candidate diagnostic biomarker in the patients with ACS [5]. Overexpression of miR-330 has a protective effect on ACS by regulating the formation of atherosclerotic plaques and vascular endothelial cell proliferation [7]. This study focused on the relationship between ACS and miR-145, which has been investigated in some cardiovascular diseases [9, 10]. The circulating expression of miR-145 was decreased in AMI and heart failure patients [9]. The reduced miR-145 that was induced by myocardial ischemia/reperfusion injury in rats could mediate the protective role of FGF21 against myocardial damage [11]. The overexpression of miR-145 has been demonstrated to serve as a potential therapeutic targets for myocardial infarction by targeting PDCD4 [12]. The differentially expressed miR-145 was closely related with the progression of acute Kawasaki disease, which has great potential to develop into ACS [13]. In addition, the regulatory role of miR-145 in cell proliferation and inflammatory response has also been reported in some other diseases [14, 15]. However, the clinical significance and functional role of miR-145 in ACS remain elusive.

In the present study, we assessed the expression patterns of miR-145 in ACS patients, as well as its diagnostic value. Additionally, the correlations of miR-145 with endothelial injury and inflammation were investigated in ACS patients and rats.

## Materials and methods

### Patients and sample collection

The present study was performed with the approval by the Ethics Committee of Yidu Central Hospital of Weifang. Serum samples were collected from 160 patients with chest pain who underwent coronary angiography in Yidu Central Hospital of Weifang from 2015 to 2017. The enrolled chest pain patients included 80 patients with ACS and 80 healthy individuals. The healthy individuals did not have coronary stenosis according to the coronary angiography, but had ≤ 2 risk factors for CAD. The diagnosis of ACS was performed in accordance with the international criteria [16–19]. The cases in the ACS patients with history of kidney disease, diabetes and severe left ventricular systolic dysfunction were excluded from this study. Venous blood samples were collected from the participants within 3–5 h of the onset of symptoms and before the arteriography. Serum was isolated from the blood samples by centrifugation and stored at − 80 °C for further analyses. All the participants wrote informed consents before the sampling. The demographic and clinical characteristics of the participants are listed in Table 1.

**Table 1:** Clinical characteristics of the participants included in this study

Characteristics	Healthy controls (*n* = 80)	ACS patients (*n* = 80)	*P* value
Age (years, mean ± SD)	55.41 ± 6.72	56.23 ± 6.11	0.712
Gender (female/male)	24/56	28/52	0.622
Risk factors (*n*)			
Hypertension	46	53	0.255
Dyslipidemia	51	64	0.022
Smoking	50	48	0.801
SBP (mmHg)	118.25 ± 20.15	122.86 ± 24.31	0.714
DBP (mmHg)	81.56 ± 16.26	81.72 ± 20.88	0.985
TC (mmol/L)	4.72 ± 0.45	4.54 ± 0.68	0.155
TG (mmol/L)	1.28 ± 0.54	1.51 ± 0.44	0.276
LDL (mmol/L)	2.57 ± 0.51	2.98 ± 0.69	0.114
HDL (mmol/L)	1.47 ± 0.52	1.32 ± 0.38	0.235
CK-MB mass (ng/mL)	0.200 ± 0.22	31.72 ± 30.26	< 0.001
Myo (ng/mL)	49.57 ± 26.31	227.25 ± 142.07	< 0.001
cTnI (ng/mL)	0.022 ± 0.035	26.42 ± 22.12	< 0.001
Subtypes			
AMI	–	42	–
UA	–	38	–

*SBP* systolic blood pressure, *DBP* diastolic blood pressure, *TC* total cholesterol, *TG* triglyceride, *LDL* low density lipoprotein, *HDL* high density lipoprotein, *CK-MB mass* creatine kinase MB mass, *Myo* myoglobin, *cTnI* cardiac troponin I, *AMI* acute myocardial infarction, *UA* unstable angina

### RNA extraction and quantitative real-time PCR (qRT-PCR)

Total RNA in the serum specimens from ACS, normal individuals and experimental rats was isolated using TRIzol reagent (Invitrogen, Carlsbad, CA, USA). Single stranded cDNA was then synthesized from the RNA by a PrimeScript RT reagent kit (TaKaRa, Shiga, Japan) following the manufacturer’s instruction. The relative expression of miR-145 was estimated by qPCR, which was carried out using a SYBR green I Master Mix kit (Invitrogen, Carlsbad, CA, USA) and a 7300 Real-Time PCR System (Applied Biosystems, USA). In the reactions, we used U6 as an endogenous control. The final expression of miR-145 was computed by 2^−ΔΔCt^ method and normalized to U6.

### ACS animal model construction and treatment

A total of 32 female Sprague–Dawley rats (weight: 200–280 g) were used for ACS modeling using coronary artery ligation as per the method previously reported [7]. In briefly, the rats were randomly divided into sham group (*n* = 8) and ACS model group (*n* = 8). The left coronary artery at about 2–3 mm from aortic root of the rats of ACS model group was found after the anesthesia by intraperitoneal injection of 1% pentobarbital sodium (40–50 mg/kg) and electrocardiograph monitoring. A 6-0 ophthalmic non-invasive suture needle was used to puncture the front and back of the left coronary vein, and the small bundle of myocardial ligation and ligation sites was observed. The chest of the rats was closed, and penicillium (200,000 U) was intraperitoneally injected to avoid infection. The electrocardiogram results were recorded, and the successfully constructed ACS model showed the elevation in ST segment and/or high T wave. The rats in sham group only received a sham operation without the ligation at coronary artery. After the ACS modeling, rats were injected with miR-145 mimic (*n* = 8) or miRNA negative control (miR-NC, *n* = 8) (GenePharma, Shanghai, China) at the myocardium near the left coronary artery to regulate the expression of miR-145 in vivo. After the treatments, serum samples were collected and kept at − 80 °C for RNA extraction to examine the expression changes of miR-145, endothelial injury biomarkers and pro-inflammatory cytokines. The experimental procedures in the animals were performed with the approval of Animal Care and Use Committee of Yidu Central Hospital of Weifang and in accordance with the Guidance for Care and Usage of Laboratory Animals.

### Enzyme-linked immunosorbent assay (ELISA)

Some endothelial injury biomarkers have been used to reflect the status of endothelial injury, such as von Willebrand factor (vWF) and heart-type fatty acid-binding protein (H-FABP) [20]. Thus, this study performed the ELISA to measure the serum concentration of vWF and H-FABP, which was carried out using an ELISA kit (Invitrogen, Carlsbad, CA, USA) as per the protocols of manufacturers. In addition, three pro-inflammatory cytokines, including interleukin-6 (IL-6) and tumor necrosis factor-α (TNF-α), were also detected in the serum samples to evaluate the change of inflammatory reaction.

### Cell culture

Rat VECs were isolated from the coronary artery tissues of each rat group using the method described in a previous study [7]. Briefly, the vascular rings were cutoff into small sections and incubated in the wells pre-coated with matrigel. The vascular sections were cultured with DMEM medium added 20% fetal bovine serum (FBS), 200 U/mL penicillin, 0.2 μg/mL streptomycin, 50 μg/mL heparin and 75 μg/mL endothelial cell growth supplement (ECGS) at 37 °C. The cells were digested using trypsin and collected when the cells grew to 80–90% confluence. Then, the cells were further digested using 0.25% trypsin to prepare the VECs suspension.

### Cell proliferation assay

Cell proliferation of the VECs was estimated using a MTT analysis. The cells were seeded into 48-well plates and cultured for 3 days. The MTT (0.5 mg/mL) was added into the cell cultures every day with 4 h further incubation. A volume of 200-μL DMSO was then added into the cells. Cell proliferation was evaluated by reading the absorbance at 490 nm by an absorbance reader (BioTek Instruments, VT, USA).

### Cell migration assay

Cell migration of the VECs was examined using Transwell chambers (Corning, USA). Cells with serum-free culture medium were added into the upper chambers and incubated at − 37 °C for 48 h. In the lower chambers, the culture medium supplemented with 10% FBS was added as attractant. Cells migrated to the lower chamber were stained after the incubation and counted using an optical inverted microscope (Nikon, Tokyo, Japan).

### Luciferase activity assay

According to the bioinformatics analysis, we predicted the potential target genes for miR-145. Forkhead box O1 (FOXO1) was found to have the complementary sequence of miR-145 at the 3′-UTR. To verify the target gene, a luciferase activity assay was performed. First, the wild-type (WT) and the mutant-type (MUT) 3′-UTR of FOXO1 were separately combined into the pMIR-Report luciferase vector (RiboBio, Guangzhou, China). Second, the VECs were co-transfected with the recombined pMIR report plasmid or the pMIR-control and miR-145 mimic or miR-NC using Lipofectamine 2000 (Invitrogen, Carlsbad, CA, USA) following the protocols of manufacturers. The luciferase activity under different treatments was examined by a Dual-Luciferase Reporter Assay (Beyotime, Jiangsu, China).

### Statistical analysis

All the data in this study were performed as mean ± SD and analyzed using the SPSS 18.0 software (SPSS Inc., Chicago, IL) and GraphPad Prism 5.0 software (GraphPad Software, Inc., USA). Comparisons between different groups were assessed using Student’s t-test and one-way ANOVA. Pearson correlation test was adopted to analyze the correlation of miR-145 with endothelial injury markers or inflammatory cytokines. In addition, a receiver operating characteristic analysis (ROC) was used to evaluate the diagnostic value of miR-145 in the patients with ACS. A difference with a *P* < 0.05 was considered as statistically significant.

## Results

### Baseline clinical characteristics of the participants in this study

The demographic and clinical characteristics of the ACS patients and healthy controls are listed in Table 1. The enrolled ACS patients included 42 cases with AMI and 38 cases with UA. The comparison results indicated that there was no significant difference between the two groups in age, gender, history of hypertension and smoking, systolic blood pressure (SBP), diastolic blood pressure (DBP), total cholesterol (TC), triglyceride (TG), low-density lipoprotein (LDL) and high-density lipoprotein (HDL) (all *P* > 0.05), but more patients with dyslipidemia were found in ACS group compared with healthy controls (*P* = 0.022), and the creatine kinase MB mass (CK-MB mass), myoglobin (Myo) and cardiac troponin (cTnI) of ACS patients were higher than that of healthy controls (all *P* < 0.001).

### Serum expression of miR-145 in the patients with ACS

**Fig. 1:**
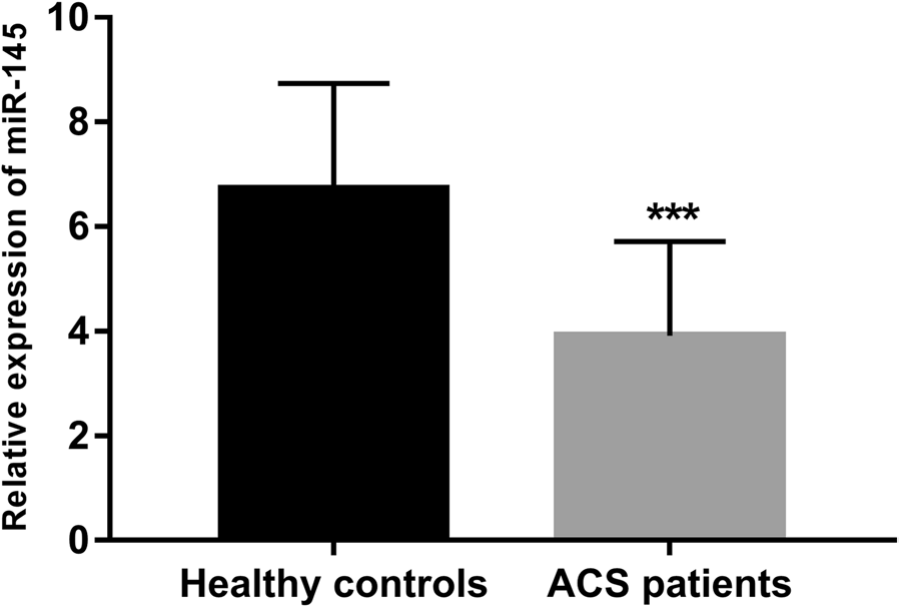
Serum expression of miR-145 was decreased in the patients with ACS compared with the healthy controls. ***P < 0.001

According to the results of qRT-PCR, we observed that the serum expression of miR-145 was significantly downregulated in the patients with ACS compared with the healthy controls (*P* < 0.001, Fig. 1).

### Diagnostic accuracy of miR-145 in the patients with ACS

**Fig. 2:**
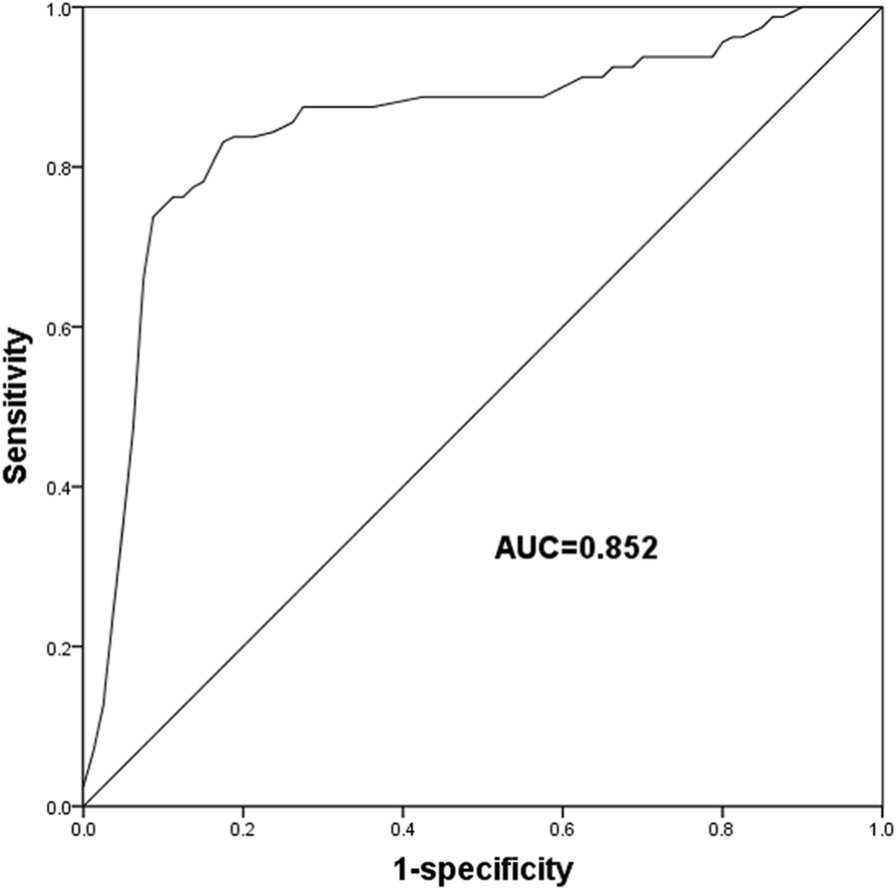
ROC curve based on the expression of miR-145. Serum expression of miR-145 had relative high diagnostic accuracy with an area under the curve (ACU) of 0.852

Since an obvious decrease in miR-145 expression was found in ACS patients, we further evaluate the diagnostic ability of miR-145 to distinguish the ACS patients from the healthy individuals. A ROC curve constructed using the serum expression levels of miR-145 revealed that the decreased expression of miR-145 had relative high diagnostic accuracy with an area under the curve (AUC) of 0.852 (Fig. 2), and the sensitivity and specificity, respectively, were 83.8% and 82.5% at the cutoff value of 5.600.

### Correlations of miR-145 with endothelial injury and inflammation in the patients with ACS

**Fig. 3:**
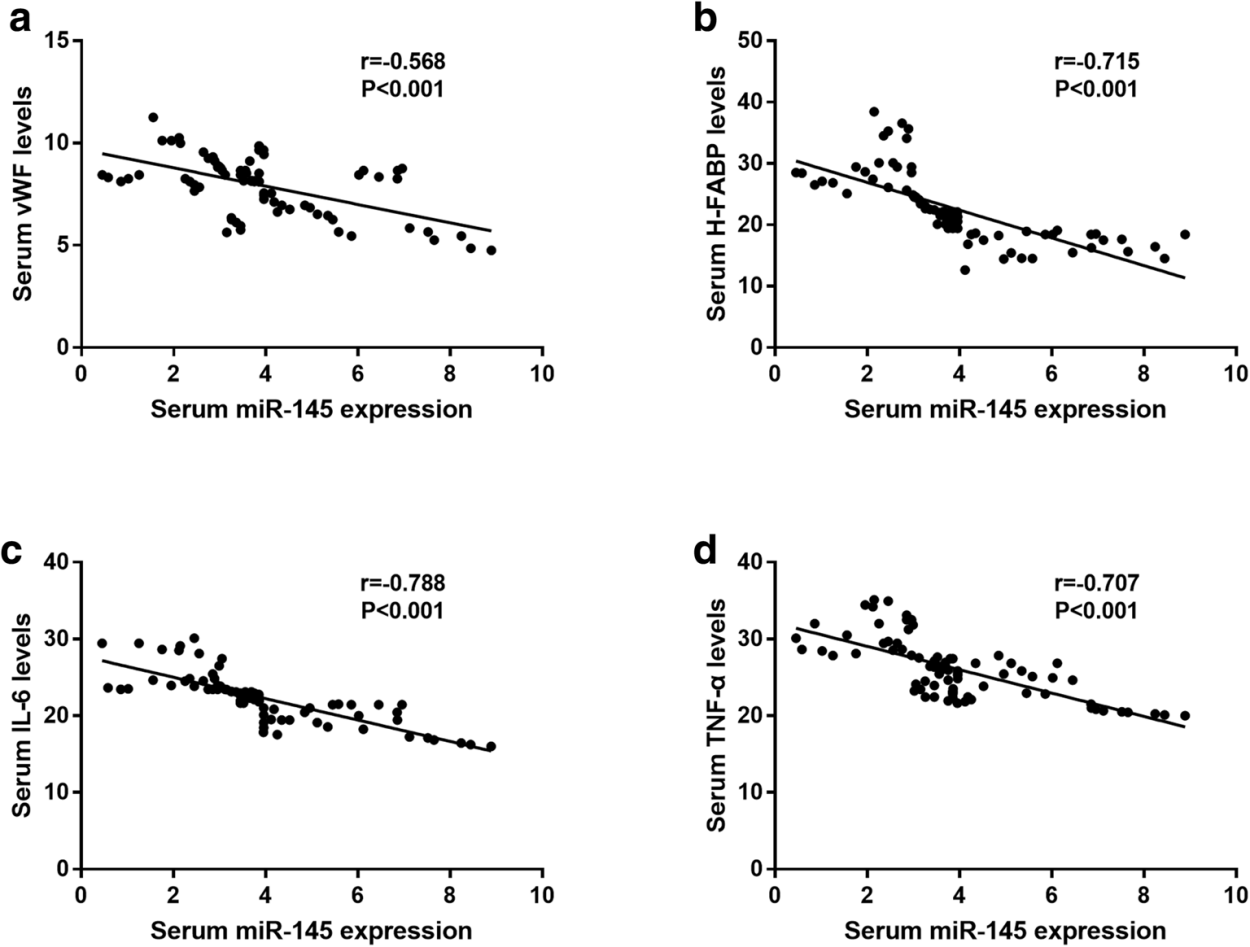
Correlation of miR-145 with endothelial injury markers and pro-inflammatory cytokines in the patients with ACS. The serum expression of miR-145 was negatively correlated with vWF, H-FABP, IL-6 and TNF-α (all *P* < 0.001)

The markers of endothelial injury and pro-inflammatory cytokines were examined as the endothelial injury and inflammation represent two key events during the progression of ACS. As shown in Table 2, we found that the serum concentrations of endothelial injury biomarkers, including vWF and H-FABP, were all elevated in the patients with ACS compared with the healthy controls (all *P* < 0.01). In addition, the markedly increases in the levels of IL-6 and TNF-α were also observed in the ACS cases compared with the healthy controls (all *P* < 0.001). Furthermore, the correlation of miR-145 expression with the levels of injury marker and pro-inflammatory cytokine was analyzed. The results shown in Fig. 3 indicated that the serum expression of miR-145 was negatively correlated with the serum levels of vWF (*r* = − 0.568, *P* < 0.001), H-FABP (*r* = − 0.715, *P* < 0.001), IL-6 (*r* = − 0.788, *P* < 0.001) and TNF-α (*r* = − 0.707, *P* < 0.001), suggesting that the aberrant expression of miR-145 might be involved in the disfunction of VECs and deregulated inflammation.

**Table 2:** Serum concentration of endothelial injury markers and pro-inflammatory cytokines

Parameters	Healthy controls	ACS patients	*P* value
vWF (ng/mL)	3.94 ± 1.70	7.93 ± 1.41	0.007
H-FABP (ng/mL)	3.73 ± 0.99	22.57 ± 5.64	< 0.001
IL-6 (ng/L)	9.28 ± 3.45	23.18 ± 5.58	< 0.001
TNF-α (ng/L)	9.21 ± 3.01	27.44 ± 5.31	< 0.001

*vWF* von Willebrand factor, *H-FABP* heart-type fatty acid-binding protein, *IL-6* interleukin-6, *TNF-α* tumor necrosis factor-α

### Effects of miR-145 on endothelial injury and inflammation in ACS rats

**Fig. 4:**
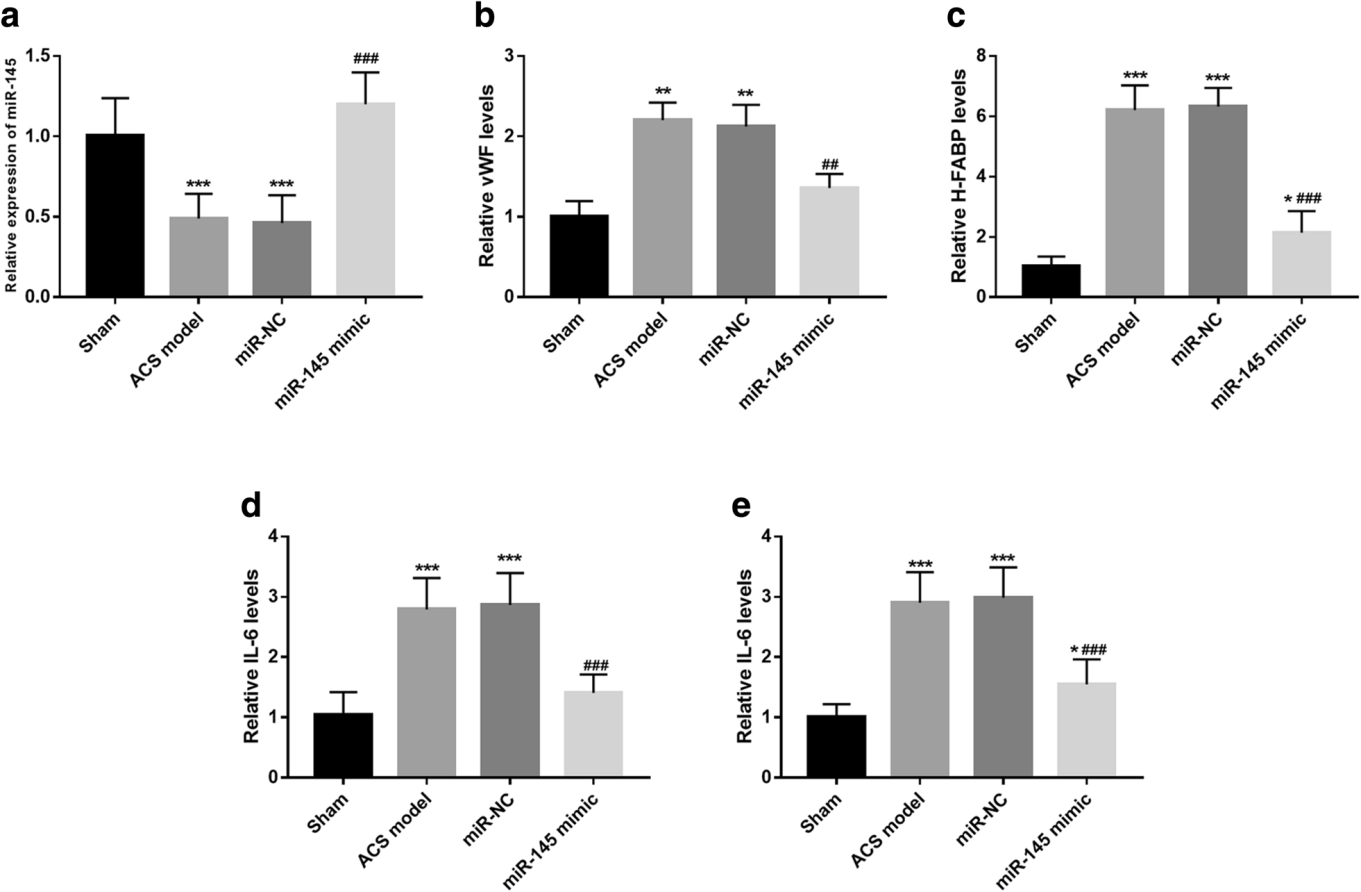
Effects of miR-145 on endothelial injury and inflammation in ACS rats. A. Serum expression of miR-145 was decreased in the ACS animal models, but was promoted by the overexpression of miR-145. **b**–**e** Serum elevated vWF (**b**), H-FABP (**c**), IL-6 (**d**) and TNF-α (**e**) in ACS rats were all abrogated by the upregulation of miR-145. *P < 0.05, **P < 0.01, ***P < 0.001, compared with the sham group; ^##^*P* < 0.01, ^###^*P* < 0.001, compared with the ACS model group

To further confirm the role of miR-145 in endothelial injury and abnormal inflammation of ACS, the ACS rat model was constructed. In accordance with the results in the ACS patients, the expression of miR-145 was also lower in the ACS rat model than that in the rats of sham group (*P* < 0.001, Fig. 4a). According to the transduction with miR-145 mimic in vivo, the expression of miR-145 in the ACS animals was successfully upregulated (*P* < 0.001, Fig. 4a). By the examination of serum endothelial injury markers and inflammatory cytokines, we found that the increased levels of vWF, H-FABP, IL-6 and TNF-α arose from ACS modeling surgery were notably decreased by the overexpression of miR-145 (all *P* < 0.01, Fig. 4b–e).

### Effects of miR-145 on cell proliferation and migration of VECs

**Fig. 5:**
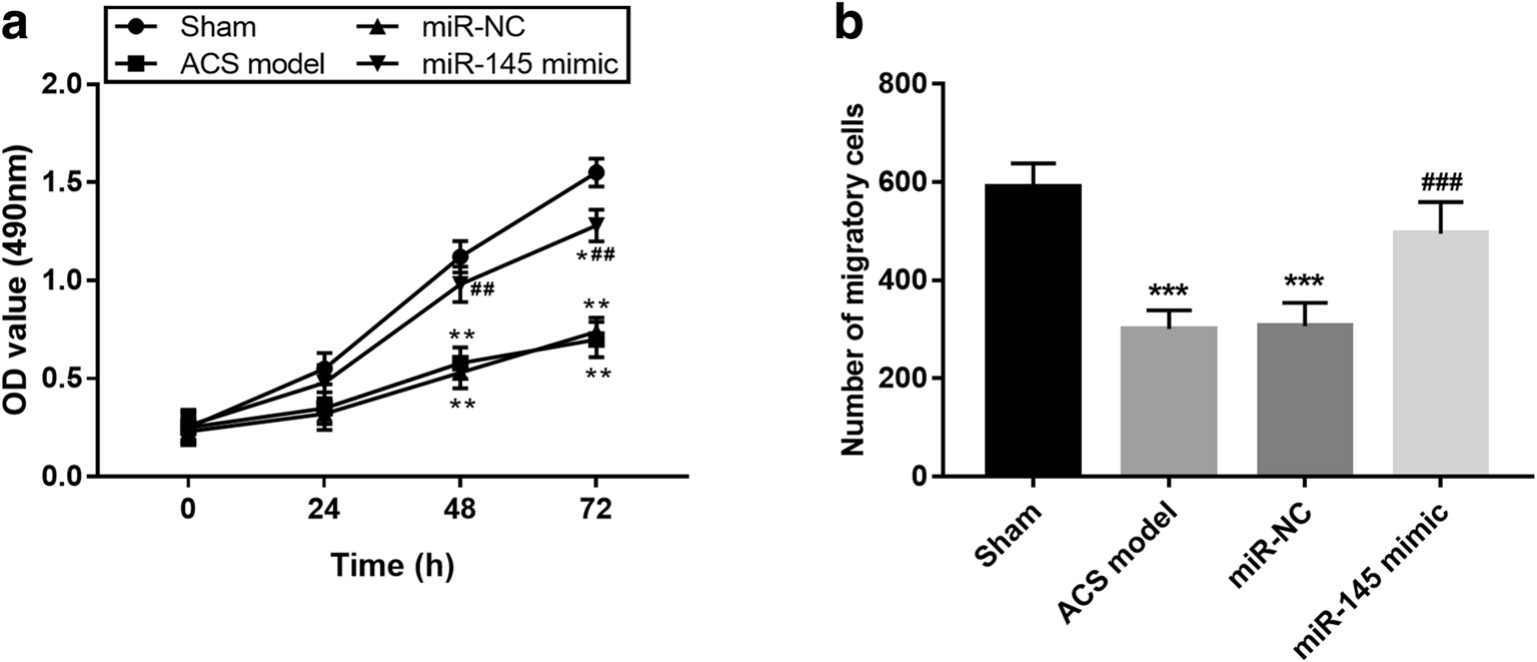
Effects of miR-145 on VECs proliferation and migration. Cell proliferation (**a**) and migration (**b**) of VECs were promoted by the overexpression of miR-145. *P < 0.05, **P < 0.01, ***P < 0.001, compared with the sham group; ^##^*P* < 0.01, ^###^*P* < 0.001, compared with the ACS model group

Cell proliferation and migration of VECs have been reported to be suppressed during the development of ACS. Thus, we focused on the effects of miR-145 on VECs proliferation and migration. The cell proliferation of VECs in the ACS model was inhibited when compared to the sham group, but was rescued by the upregulation of miR-145 (all *P* < 0.05, Fig. 5a). Similarly, the blocked cell migration in VECs was abrogated by the overexpression of miR-145 (all *P* < 0.001, Fig. 5b).

### FOXO1 serves as a target gene of miR-145

**Fig. 6:**
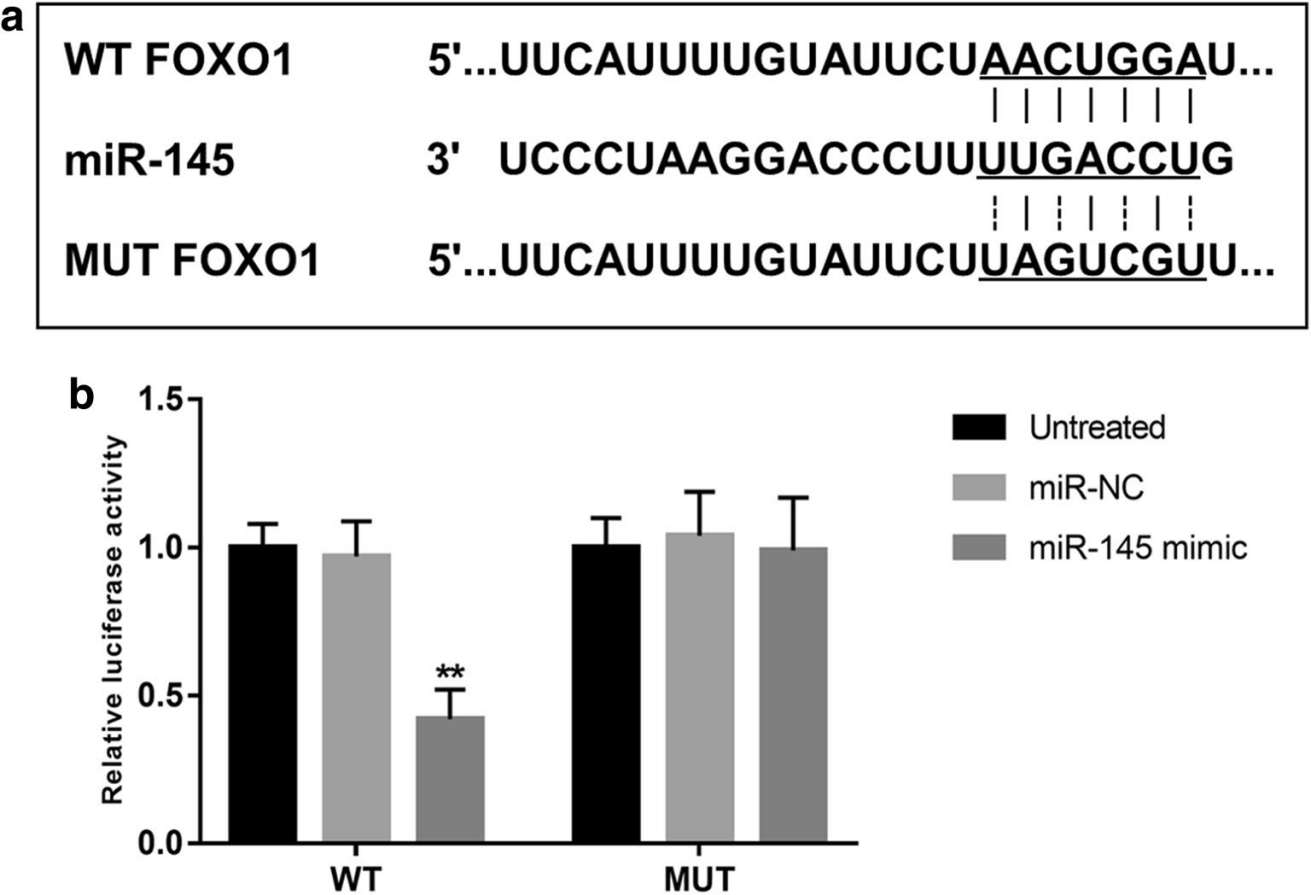
FOXO1 serves as a target gene of miR-145. **a** The complementary sequence of miR-145 in the 3′-UTR of FOXO1. **b** Luciferase activity of the WT group was suppressed by the overexpression of miR-145. **P < 0.01

To further understand the molecular mechanisms of miR-145 acting in ACS, the potential targets of miR-145 were predicted in this study. In the 3′-UTR of FOXO1, we found a complementary sequence for miR-145 (Fig. 6a). The luciferase report assay results indicated that the relative luciferase activity in the WT group was significantly suppressed by the overexpression of miR-145 (*P* < 0.01, Fig. 6b). However, no changes were observed in the luciferase activity in the MUT group.

## Discussion

Emerging studies highlight the critical roles of miRNAs in various human disease, including cardiovascular diseases [21]. For example, the increased circulating miR-195-3p was demonstrated to be a diagnostic biomarker in the patients with heart failure [22]. miR-487b has the ability to improve chronic heart failure by reducing the myocardial apoptosis and inflammatory response through the IL-33/ST2 signaling pathway [23]. The deregulated miR-362-3p was involved in the regulation of vascular smooth muscle cell proliferation and migration in atherosclerosis according to downregulating ADAMTS1 [24]. In ACS, there are also some functional miRNAs associated with the development of this disease. Bai et al. [5] reported that plasma elevated expression of miR-941 served a diagnostic biomarker in the patients with ACS. Ren et al. [7] found that the upregulation of miR-330 could inhibit the formation of atherosclerotic plaques and facilitate VECs proliferation by targeting MAPK8 via the WNT signaling pathway. All these aforementioned studies indicated that the functional miRNAs have the potentials to improve the diagnosis and treatment of ACS.

During the development of ACS, impairments in the function of VECs and the inflammatory response are considered as two important events. Some studies regarding the improvement of the two events have been conducted for the treatment of ACS [25]. For instance, miR-150 has been reported to ameliorate ACS by restoring the function of VECs, such as cell proliferation and migration [26]. Darabi et al. [27] indicated that the elevated expression of miR-21 might be associated with the pathogenesis of ACS by regulating inflammation. In this study, we found that the serum expression of miR-145 was significantly decreased in the patients with ACS compared with the healthy controls. More importantly, the aberrant expression of miR-145 was negatively correlated with the serum levels of endothelial injury biomarkers and pro-inflammatory cytokines. Thus, we hypothesized that the dysregulation of miR-145 might be involved in the progression of ACS by regulating the function of VECs and inflammation. However, information about the patients’ characteristics is not sufficient enough, for example co-morbidities, laboratory parameters and co-medication were lacking. More studies with more comprehensive collection of patients' characteristics are needed to verify the present results.

miR-145 has been investigated in some other human diseases, such as malignancy [28], diabetes [29] and also other cardiovascular disease [30]. The functional role of miR-145 in the diseases above has been uncovered. In addition, the clinical significance of miR-145 has also been explored and discussed in some disease. For example, the decreased miR-145 expression was demonstrated to serve as a prognostic biomarker in the patients with gastric cancer [31]. Plasma miR-145 expression was determined as a diagnostic biomarker for the differentiation of cervical cancer cases from healthy individuals [32]. Given the decreased expression of miR-145 in ACS patients, we investigated its diagnostic value using the ROC analysis. The results indicated that the expression of miR-145 had relative high diagnostic accuracy to distinguish ACS patients from the healthy controls.

To further confirm the functional role of miR-145 in the dysfunction of VECs and abnormal inflammation of ACS, the ACS rat model was constructed using coronary artery ligation. Similar expression profile of miR-145 in ACS patients was also observed in the ACS rats, indicating the important role of miR-145 in the pathogenesis of ACS. By regulating the expression of miR-145 in vivo, we found that the overexpression of miR-145 could improve the function of VECs evidenced by the decreased concentration of endothelial injury markers and the promoted cell proliferation and migration. Meanwhile, the deregulated inflammatory cytokines in ACS rats were all suppressed by the upregulation of miR-145, indicating the beneficial effects of miR-145 on the inflammation of ACS. These study used vWF and H-FABP as endothelial injury biomarkers and IL-6 and TNF-α as representative pro-inflammatory cytokines, which have been widely used to evaluate the injury condition of endothelial function and inflammation status in previous studies [33, 34]. However, further studies are needed to confirm the regulatory effects of miR-145 by detecting more molecular markers or using more intuitive detection methods. Although this study provided evidence for miR-145 to reduce ACS-related endothelial injury and inflammatory responses, the expression changes of miR-145 in myocardium were not examined, leading to the limited understand about the mechanisms underlying the inhibiting effect of miR-145 on inflammation and endothelial injury. Considering the increase in circulating miR-145 expression, inflammatory cells might engulfed miR-145 then depolarized, and thus leading to the non-inflammatory nature and beneficial effect on endothelial cells. This hypothesis warrants to be confirmed in further investigations. Another limitation of the present study was that the effect of miR-145 on infarct size in ACS rats was not analyzed. Further studies should focus on more pathological changes under the dysregulation of miR-145 to confirm the role of miR-145 in ACS.

The effects of miR-145 on cell biological function and inflammation have been investigated in some diseases. In gastric cancer, miR-145 could inhibit the tumor cell migration by targeting MYO6 [28]. In neuropathic pain, miR-145 could ameliorate this perplex by inhibiting inflammatory responses through the mTOR signaling pathway [35]. In the present study, we predicted the target gene of miR-145, and proved that FOXO1 was a target gene in VECs. A study by Jiang et al. [36] has reported that miR-145 regulated tumor cell growth by targeting FOXO1. The osteogenic differentiation could be regulated by miR-145 through targeting FOXO1 [37]. Moreover, the effects of FOXO1 on cell biological function and inflammatory reaction have been previously reported [38, 39]. Therefore, we considered that the regulatory effects of miR-145 on VECs proliferation and migration and inflammation in ACS might be achieved by targeting FOXO1. A previous study by Hu et al. has reported that FGF21 had a protective effect on myocardial ischemia–reperfusion injury by regulating miR-145 [11]. The dysregulation of FGF21 has been found in AMI patients and might be involved in the pathogenesis of AMI [40–42]. However, whether FGF21 also affected endothelial injury and inflammation by the regulation of miR-145 in ACS has not been investigated. Thus, the precise molecular mechanisms underlying the role of miR-145 need to be confirmed and analyzed in further studies.

## Conclusion

Taken together, the results of this study revealed that the decreased expression of miR-145 plays a promising diagnostic biomarker for the patients with ACS. The overexpression of miR-145 may contribute to the recovery of VECs function and inflammation by downregulating FOXO1. Thus, it was hypothesized that the methods to promote miR-145 expression may have great potentials to improve the treatment of ACS.

## Data Availability

All data generated or analyzed during this study are included in this published article.

## Abbreviations

ACS: Acute coronary syndrome
miR-145: microRNA-145
VECs: Vascular endothelial cells
AMI: Acute myocardial infarction
UA: Unstable angina
miR-145: microRNA-145
miRNAs: microRNAs
qRT-PCR: Quantitative real-time PCR
miR-NC: miRNA negative control
ELISA: Enzyme-linked immunosorbent assay
vWF: von Willebrand factor
H-FABP: Heart-type fatty acid-binding protein
IL-6: Interleukin-6
TNF-α: Tumor necrosis factor-α
FBS: Fetal bovine serum
ECGS: Endothelial cell growth supplement
